# Drowned in Dust

**Published:** 1898-09-24

**Authors:** 


### DROWNED IN DUST.

According to the New York *Medical Record,* a ranchman in Iowa recently met his death from a curious cause, a cause which, if the recent drought had not been interrupted by a few welcome showers, might well have operated in some parts of England. The man had left the town for his home, which was some miles distant, in the hottest part of the day. A mile from town he was overcome by the heat, and possibly by his potations, and fell from his horse. The dust was at this place several inches deep, and very fine; he fell on his face and was suffocated. The verdict of the jury was that he was "smothered to death in the dust in the public highway."

